# Editorial: Microbial diversity and ecosystem functioning in fragmented rivers worldwide

**DOI:** 10.3389/fmicb.2023.1253190

**Published:** 2023-11-08

**Authors:** Lunhui Lu, Dayong Zhao, Zhe Li, Leonardo D. Fernández

**Affiliations:** ^1^CAS Key Laboratory of Reservoir Water Environment, Chongqing Institute of Green and Intelligent Technology, Chinese Academy of Sciences, Chongqing, China; ^2^State Key Laboratory of Hydrology-Water Resources and Hydraulic Engineering, Joint International Research Laboratory of Global Change and Water Cycle, Hohai University, Nanjing, China; ^3^Centro de Investigación en Recursos Naturales y Sustentabilidad (CIRENYS), Universidad Bernardo O'Higgins, Santiago, Chile

**Keywords:** fragmentation, damming rivers, microbial diversity, ecosystem functionality, community assembly

Rivers are the cradle of human society. The water resource system is essential for human reproduction and development as a complex social, economic, and ecological system. Building dams and reservoirs has a significant effect in regulating freshwater resources in a watershed, providing various functions and ecosystem services such as irrigation, hydropower generation, flood control, water supply, aquaculture, navigation, and tourism to serve the economic development of human society. Dam construction and reservoir formation are probably the most significant anthropogenic footprints on water cycling and river ecosystems.

Since the 1960s, some viewpoints have suggested that the negative effects of dam construction on river ecosystems outweigh their positive effects, leading to irreversible loss of species and ecosystem functions in many cases. Dam construction and impoundment change rivers from “lotic systems” to “lentic systems,” leading to different hydrological characteristics in the river-reservoir region, and changing the element cycling and the riverine aquatic ecosystem. Microbial diversity, co-occurrence network interaction, and community assembly are crucial indicators driving the ecosystem functionality and process. Planktonic microorganisms in the river ecosystem fundamentally and significantly regulate and maintain the ecological structure and functioning.

This Research Topic “*Microbial diversity and ecosystem functioning in fragmented rivers worldwide*,” which includes 15 original research articles, mainly focuses on microbial diversity, microbial community assembly mechanisms, functional genes, and microbial interaction in damming rivers and reservoirs worldwide. The research objectives include phytoplankton, planktonic bacteria, eukaryotic microorganisms, archaea, functional microorganisms such as methanogens, etc. In addition, macroinvertebrates as the important indicators of ecosystem functionality are also considered in our Research Topic. The study area and habitats include large rivers, reservoirs, lakes, small streams, and riparian zones. The research techniques include but are not limited to microbial community structure and function, network interaction, community assembly process, microbial traceability, microbial carbon metabolism, and ecosystem function. Planktonic microorganisms are classified as free-living and particle-attached in the aquatic systems according to their living habitats. One article studied free-living and particle-attached bacterial communities in the Wujiangdu river reservoir of the Yungui Plateau, China. The free-living and particle-attached bacteria showed distinct communities and diversity characteristics of bacterial fractions. This result indicates that the suspended particles in river-reservoir systems are important carriers and living environments for the planktonic microorganisms. Another research article from Yu et al. also made research on particle-attached and free-living bacteria in a reservoir. In addition to analyzing the microbial network interaction and community assembly of these two bacterial fractions, the microbial functional assemblages, especially for the putative genetic capacity of nitrogen and carbon cycling function, were annotated using FAPROTAX_1.1. The effect of damming rivers on ecosystem function is a controversial topic. Researchers from Tianjin University proposed a function-to-taxa ratio (F:R) to estimate the stability based on functional redundancy theory, and results indicate that river damming in the Pearl River Basin enhances the ecological functional stability of planktonic microorganisms. In addition, archaea were indicated to contribute more carbon function and play a predominant ecological process in sediment. In addition to macroinvertebrates, there are strong longitudinal gradient differentiations of *Daphnia galeata* populations in southern China reservoirs, and the deterministic process of environmental selection was the main community assembly process. Rivers are fragmented by different barriers. Here, Xing et al. conducted a study in different habitats of Liangtan River, Chongqing, China. Results indicate different barrier heights and habitats may lead to different greenhouse gas emissions processes at the water-air interface. Microbial community assembly processes and co-occurrence network analyses were important to measure the ecological processes of the community and are usually estimated by a null model. Another study conducted in Taihu Lake indicated that rare denitrifying anaerobic methane-oxidizing bacteria (DAMO bacteria) communities were mainly shaped by deterministic processes. Agricultural fertilizer was identified as the main primary contamination source through microbial source tracking technology, indicating a new precise method for pollutant sourcing compared with traditional chemical methods. The mid-channel bar was easily impacted by the hydrological runoff. Co-occurrence network hubs of the rhizosphere fungal communities in the mid-channel bar of the Yangtze River are the key species linking other species in the communities. The microbial community assembly processes were distinct between the soil and river ecosystems. The rhizosphere fungal community assembly in the mid-channel bar of the Yangtze River was mainly shaped by stochastic processes. Like the mid-channel bar, the riparian zones were important transitional areas between aquatic and terrestrial ecosystems, and microbial activity may be disturbed by water level fluctuations. Microbial carbon efficiency (CUE) can reflect the soil carbon storage capacity, and this study described microbial carbon use efficiency and microbial biomass carbon patterns in the Three Gorges Reservoir. This study can give a potential prediction of carbon cycling and microbial carbon pump mechanism in aquatic-terrestrial ecotones.

The research results collected in this Research Topic are not comprehensive. In summary, this Research Topic collected diverse results about the microbiome in different habitats such as large rivers, reservoirs, lakes, small streams, and riparian zones. These collected manuscripts in this Research Topic provided some directions for the study of microbiomes in the river-reservoir ecosystem. Nevertheless, a comprehensive study of microbial ecology in river ecosystems is still needed due to the complexity and variability of the water ecosystem. Hence, future research should focus on microbial processes mechanisms, such as the immunity, stability, and resilience of aquatic ecosystems under changing hydrological environments. It is also very important to develop adaptive river management strategies to promote the healthy and sustainable development of river ecosystems. In addition, from the perspective of global river damming, the impact of different dams in different climatic regions on water ecology is needed. New perspectives are also needed to provide a deep understanding of the ecological effects of river fragmentation.

## Author contributions

All authors listed have made a substantial, direct, and intellectual contribution to the work and approved it for publication.

